# Burden and Trends of Low Back Pain in Pakistan: A Global Burden of Disease Study, 1990–2021

**DOI:** 10.7759/cureus.93105

**Published:** 2025-09-24

**Authors:** Muhammad Tayyab, Shah Faisal, Zawar Ahmad, Mahmood Ahmad, Muhammad Tanveer, Suleman Shah, Mohammad Owais Ali Shah, Ameer Afzal Khan, Rahman Syed, Mohsin Ali, Anfal Khan, Muhammad Shabir, Muhammad Rabbani

**Affiliations:** 1 Department of Trauma and Orthopaedics, Bradford Teaching Hospitals, Bradford, GBR; 2 Department of Orthopaedics, Saidu Group of Teaching Hospitals, Swat, PAK; 3 Department of Trauma and Orthopaedics, Kettering General Hospital, Kettering, GBR; 4 Department of Trauma and Orthopaedics, Milton Keynes University Hospital, Milton Keynes, GBR; 5 Department of Trauma and Orthopaedics, Royal Stoke University Hospital, North Midlands, GBR; 6 Department of Nursing, Fatima College of Health Sciences, Al Ain, ARE; 7 Department of Internal Medicine, Saidu Medical College, Swat, PAK; 8 Department of Internal Medicine, Swat Medical College, Swat, PAK

**Keywords:** global burden of disease (gbd), low back pain, pakistan, prevalence, ylds

## Abstract

Background and aim

Low back pain (LBP) is a major contributor to disability globally, yet national and provincial trends in Pakistan remain inadequately explored. This study aimed to quantify the national and provincial trends in the prevalence of low back pain (LBP) in Pakistan from 1990 to 2021 using the Global Burden of Disease (GBD) 2021 data, assess the trends in years lived with disability (YLDs) attributable to LBP over the same period, and identify temporal changes using Joinpoint regression and highlight regional and gender disparities in the burden of LBP.

Methods

Age-standardized rates of prevalence and YLDs per 100,000 population due to LBP were analyzed for Pakistan and its provinces. Temporal patterns were evaluated using Joinpoint regression analysis to estimate the annual percent changes (APC) with 95% confidence intervals.

Results

In 1990, the prevalence of LBP in Pakistan was 6,500 (95% uncertainty interval (UI): 5,466-7,638) per 100,000, which increased to 7,462 (95% UI: 6,300-8,763) in 2021, reflecting a 14.8% rise. The highest prevalence in 2021 was observed in Gilgit-Baltistan (8,302; 95% UI: 7,260-9,322), while Punjab and Islamabad Capital Territory showed the largest relative increases (+14.8% and +11.8%, respectively). YLDs also rose from 605 (95% UI: 503-713) per 100,000 in 1990 to 720 (95% UI: 609-842) in 2021. Joinpoint regression revealed four phases: a gradual increase (1990-2001; APC 0.08%, p<0.001), moderate growth (2001-2010; APC 0.27%, p<0.001), sharp escalation (2010-2015; APC 1.86%, p<0.001), and a decline thereafter (2015-2021; APC -0.44%, p<0.001).

Conclusions

The burden of LBP in Pakistan has increased substantially over the past three decades, with notable regional disparities. These findings emphasize the need for targeted preventive, clinical, and rehabilitative interventions to reduce disability related to LBP.

## Introduction

Low back pain (LBP) is a pervasive condition that goes beyond borders, cultures, and age groups, often beginning as a dull ache and evolving into a chronic cause of disability. It is one of the leading causes of years lived with disability (YLDs) worldwide, as it surpasses many other musculoskeletal disorders such as neck pain, osteoarthritis, rheumatoid arthritis, and gout in its impact on the quality of life and productivity [1]. In 2020, an estimated 619 million people were living with LBP, and projections and studies suggest that this number could rise to 840 million by 2050, with population aging and other risk factors adding their parts [2,3]. This upward surge highlights the urgency for both preventive and therapeutic strategies.

The burden of LBP varies across countries by demographics, occupations, and lifestyle factors. Women consistently report higher rates of LBP than men, and prevalence increases sharply with age, particularly after the fifth decade of life [1,4]. Globally, the middle-aged group (45-59 years) puts a significant share of the disability burden, with disability-adjusted life years (DALYs) in this group rising from about 5.5 million in 1990 to nearly 9.8 million in 2021 despite a slight decline in the DALY rate per 100,000 population [1].

Modifiable risk factors, including occupational place exposures, high body mass index (BMI), and smoking, are major contributors to the global LBP burden according to a study [5]. In 2021, these accounted for almost 39% of YLDs due to LBP worldwide [5]. The risk profile also shows a strong regional variation, with physically demanding jobs, poor ergonomic environments, and limited access to rehabilitation services intensifying the burden in low- and middle-income countries (LMICs) [2,6].

In Pakistan, LBP presents a unique challenge. The Global Burden of Disease Study in 2021 revealed a 10.48% (95% uncertainty interval (UI): 7.60-13.56%) increase in the age-standardized prevalence rate of LBP in Pakistan from 1990 to 2021, which is one of the highest increases worldwide. In contrast, China experienced a 19.49% (95% UI: -21.04 to -18.21%) decrease over the same period, while India saw a 13.69% (95% UI: -14.99 to -12.43%) decrease in age-standardized prevalence [1]. This persistent rise hints at the influence of country-specific factors such as high rates of informal labor, underdeveloped workplace safety regulations, and limited awareness about musculoskeletal health.

Among nurses in public tertiary hospitals in Rawalpindi, 72% reported experiencing LBP in the past year, with older age, longer job tenure, and heavy workloads identified as significant predictors [7]. In Punjab’s coal mining areas, specifically tasks such as coal cutting were associated with a more than 13-fold increase in the odds of developing LBP, with work experience and repetitive motion contributing further [8]. Another study on patients with LBP found that they had increased BMI, showing that it is also a risk factor [9]. LBP not only affects the spine, but it also negatively influences livelihoods, mood patterns, emotional well-being, and the ability to participate in family and community life.

Comparable trends have been reported across South Asia, where LBP remains a leading cause of disability. In India, a 2022 meta-analysis found lifetime prevalence as high as 66% (95% UI: 56%-75%), with point and 12-month prevalences of 48% and 51%, respectively [10]. In Bangladesh, a national survey for adults aged ≥18 years reported a current LBP prevalence of 18.5% (age-standardized 19.4%) while studies in specific groups (e.g. medical students) showed 12-month prevalence up to 63.3% [11,12]. In Nepal, population estimates from the World Health Organization's STEPSwise approach to Noncommunicable Disease (NCD) Risk-Factor Surveillance (WHO STEPS) survey (2019) indicate that about 23.2% of adults (15-69 years) experience activity-limiting LBP [13]. These regional data underscore the persistent and widespread impact of LBP across LMICs, warranting country‐specific analyses such as ours.

This study aims to analyze the burden and trends of LBP in Pakistan from 1990 to 2021 using GBD data to assess national patterns within the global landscape. By doing so, we hope to highlight the interplay of demographic change, occupational exposures, and lifestyle factors, and to inform authorities whose timely intervention can take steps to alleviate the risk factors for this condition.

## Materials and methods

Data source

The data for this analysis was taken from the GBD 2021 study, a major epidemiological database that assesses the burden of 371 illnesses and injuries, as well as 88 risk factors, across 204 countries and territories [14,15]. To allow for comparisons of regional and worldwide burdens, the GBD research used standardized ways to assess illness indicators such as incidence, prevalence, YLDs, and disability-adjusted life years (DALYs). In the GBD framework, DALYs are a composite measure that combines years of life lost (YLLs) due to premature mortality and YLDs. YLDs represent the non-fatal component, calculated by multiplying disease prevalence with disability weights that reflect the severity of health loss. This analysis was based on data on LBP and the YLDs associated with it in Pakistan from 1990 to 2021. Data on age-standardized prevalence and YLDs per 100,000 population were retrieved from the GBD 2021 study via the Global Health Data Exchange (GHDx) portal (http://ghdx.healthdata.org/gbd-results-tool). Provincial-level estimates were obtained from the same portal by selecting Pakistan’s subnational units (provinces/territories). All data used are publicly available and can be reproduced by following these steps.

Study design

We conducted a secondary, population-based time-trend analysis of LBP in Pakistan from 1990 to 2021 using publicly available estimates from the GBD 2021 study.

Case definition

According to GBD criteria, low back pain is defined as localized pain in the lumbar region that lasts at least a day, reaches from the inferior gluteal fold to the lower edge of the 12th rib, and may or may not radiate to the lower limbs [16].

Data processing and quality assurance

To ensure high data quality and comparability, we used the GBD 2021 database, which harmonizes and standardizes information from a variety of sources, including published research, household surveys, and vital registries [14]. Before analysis, the dataset was cleaned using multiple imputation approaches described in the GBD methodology [15] to remove duplicates, correct inconsistencies, and resolve missing data. To allow for geographical and temporal comparisons, age-standardized rates were calculated using the World Health Organization's world standard population [17].

Statistical analysis

Joinpoint regression modelling was used to investigate trends in Pakistan's age-standardized prevalence and YLD rates of LBP from 1990 to 2021 using the Joinpoint Regression Program (version 4.9.1.0; National Cancer Institute, Bethesda, MD, USA) [18]. This method calculates the annual percentage change (APC) between the points when statistically significant trend changes occur. To represent the overall trend, the average annual percentage change (AAPC) over time was also determined. The statistical significance was determined using 95% confidence intervals and a 5% alpha threshold.

Ethical considerations

This study is based on secondary analysis of publicly available de-identified data from the GBD database and does not require institutional ethical approval.

## Results

Prevalence of low back pain

The number of prevalent cases of LBP in Pakistan increased markedly between 1990 and 2021. In 1990, an estimated 5.23 million cases were reported (95% UI: 4.52-6.07 million), which rose to 13.93 million cases in 2021 (95% UI: 12.01-16.06 million), representing a 166% increase over the 31 years. Part of this rise reflects Pakistan’s rapid population growth during the study period from approximately 107 million in 1990 to over 225 million in 2021, which substantially increased the absolute number of people at risk.

**Figure 1:**
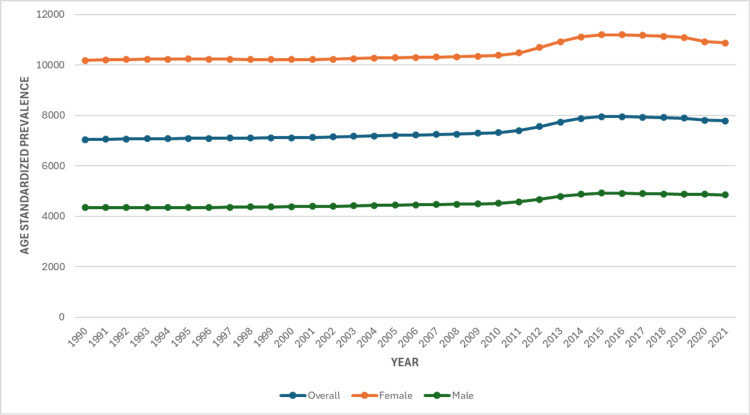
Age-Standardized Prevalence of Low Back Pain in Pakistan, from 1990 to 2021

The age-standardized prevalence rate was 7,048.51 per 100,000 population (95% UI: 6,070.55-8,089.97) in 1990, with a significantly higher burden among women (10,188.21; 95% UI: 8,780.00-11,670.70) compared with men (4,345.05; 95% UI: 3,693.21-4,979.61). By 2021, the overall prevalence rate had increased to 7,787.21 per 100,000 population (95% UI: 6,686.41-8,936.80), corresponding to a 10.5% rise. During this period, the female prevalence rate increased to 10,883.43 (95% UI: 9,362.13-12,441.76), a 6.8% increase, while the male prevalence rose to 4,848.62 (95% UI: 4,110.60-5,574.27), reflecting an 11.6% increase, as shown in Figure 1.

**Figure 2:**
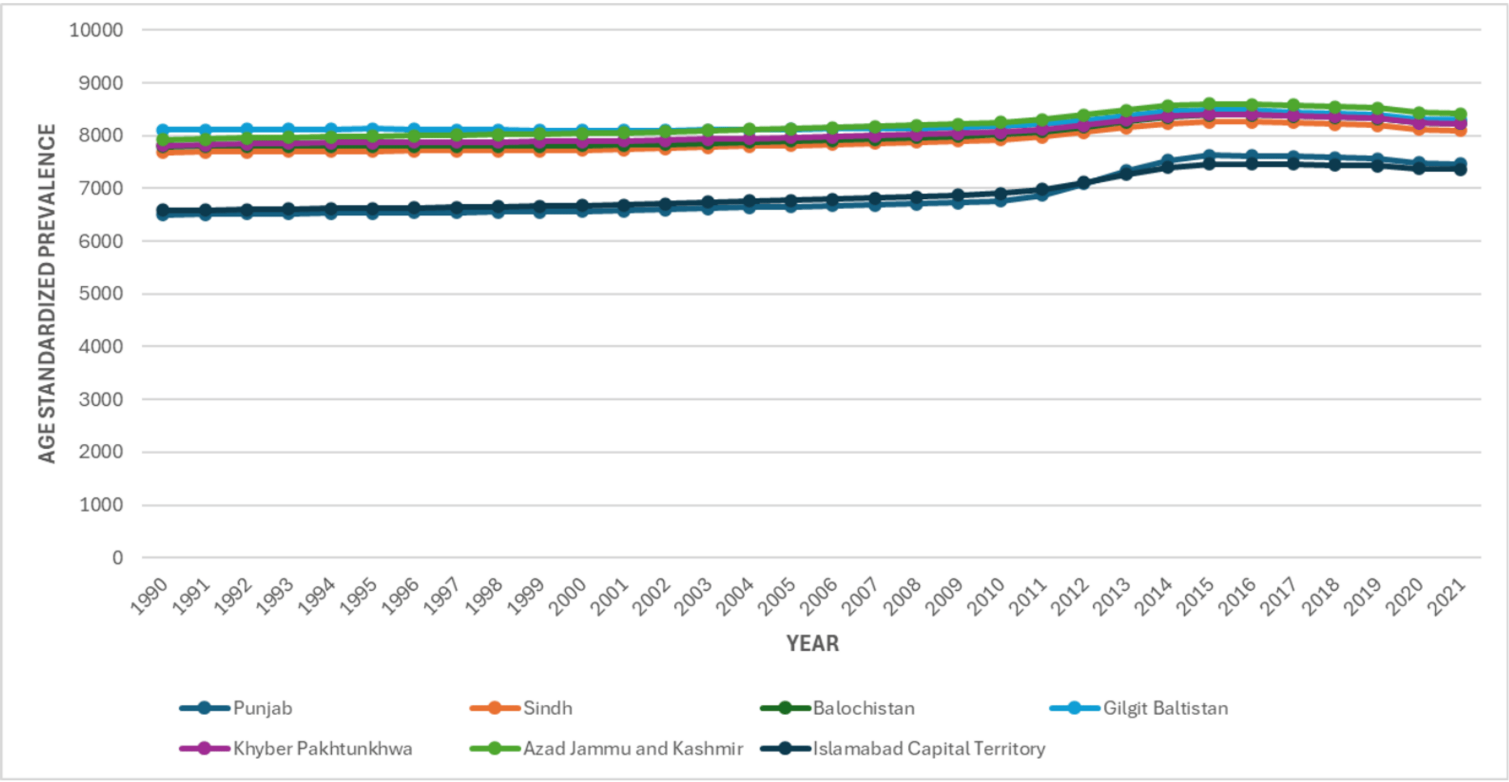
Age-Standardized Prevalence of Low Back Pain in the Provinces of Pakistan from 1990 to 2021

In 1990, the age-standardized prevalence of LBP per 100,000 population was highest in Gilgit-Baltistan at 8,107 (95% UI: 7,080-9,121), followed closely by Khyber Pakhtunkhwa at 7,814 (6,849-8,818) and Balochistan at 7,794 (6,835-8,755). The lowest rates were observed in Punjab at 6,500 (5,466-7,638) and the Islamabad Capital Territory (ICT) at 6,583 (5,474-7,632). By 2021, the prevalence of LBP had risen across all provinces and territories. Gilgit-Baltistan remained the highest-burdened region with 8,302 cases (7,260-9,322), although this represented the smallest percent change (+2.4%) over the study period. Other provinces with high baseline prevalence demonstrated modest increases, including Sindh (+5.3% to 8,097: 95% UI, 7,097-9,097), Khyber Pakhtunkhwa (+5.3% to 8,226: 95% UI, 7,163-9,181), and Balochistan (+5.7% to 8,234: 95% UI, 7,210-9,339). In contrast, regions with initially lower prevalence exhibited the greatest relative increases. Punjab showed the largest rise (+14.8%), reaching 7,462 (6,300-8,763), while ICT increased by +11.8% to 7,361 (6,137-8,600). Azad Jammu & Kashmir also demonstrated a moderate increase (+6.3%), reaching 8,420 (7,351-9,432), as shown in Figure 2.

Years lived with disability (YLDs)

**Figure 3:**
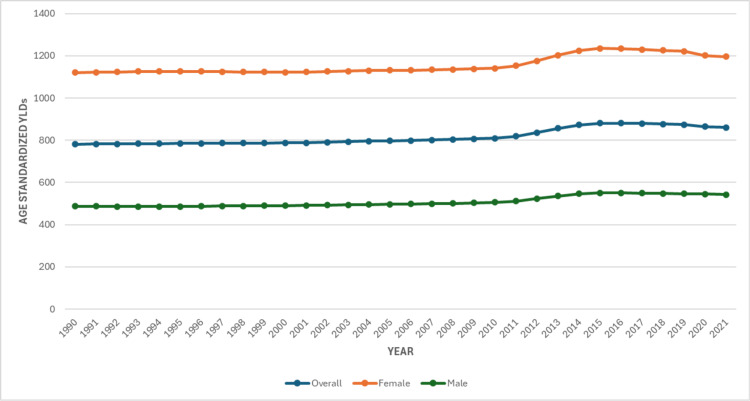
Age-Standardized Years Lived with Disability (YLD) of Low Back Pain in Pakistan, from 1990 to 2021

In 1990, the age-standardized YLD rate for low back pain in Pakistan was 781.04 per 100,000 population (95% UI: 553.26-1,059.82). By 2021, this had increased to 861.00 per 100,000 population (95% UI: 604.05-1,170.82), representing a 10.2% rise. The YLD burden peaked in 2015 at 881.25 (95% UI: 617.11-1,194.61) before showing a modest decline in subsequent years, as shown in Figure 3.

A persistent gender disparity was observed throughout the study period. Among women, the YLD rate increased from 1,121.24 (95% UI: 802.32-1,519.55) in 1990 to 1,195.75 (95% UI: 846.91-1,623.98) in 2021, a 6.6% increase. In contrast, men experienced a sharper relative rise, increasing from 487.15 (95% UI: 345.44-674.49) to 542.89 (95% UI: 385.42-733.11), an 11.4% increase, as shown in Figure 3.

**Figure 4:**
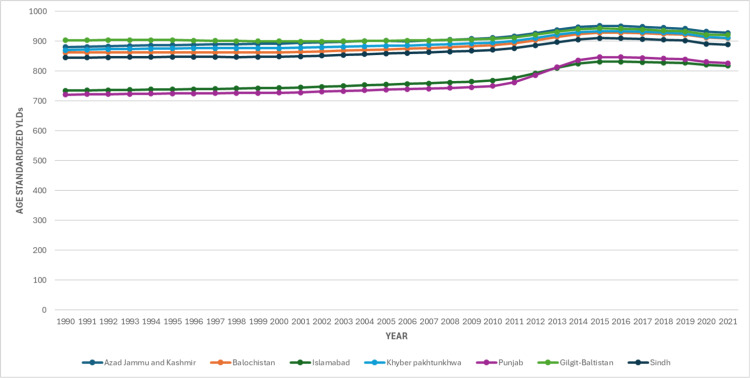
Age Standardized Years Lived with Disability (YLD) of Low Back Pain in the Provinces of Pakistan, from 1990 to 2021

Subnational analyses revealed considerable heterogeneity in the burden of LBP across Pakistan. In 2021, the highest age-standardized YLD rate was observed in Gilgit-Baltistan (920.88; 95% UI: 650.91-1,246.49), showing only a marginal increase from 1990 (903.12; 95% UI: 638.24-1,218.01), equivalent to a 2.0% rise. By contrast, Punjab consistently recorded the lowest rates, although it exhibited a substantial increase over the study period, rising from 721.03 (95% UI: 500.02-994.71) in 1990 to 826.85 (95% UI: 575.65-1,131.42) in 2021, representing a 14.7% rise. Other provinces demonstrated intermediate increases, with Khyber Pakhtunkhwa rising from 870.49 to 911.62 (4.7% increase), Sindh from 845.15 to 888.89 (5.2% increase), Balochistan from 862.32 to 910.17 (5.5% increase), and the Islamabad Capital Territory from 734.83 to 817.65 (11.3% increase), as shown in Figure 4.

Temporal trends in prevalence

Joinpoint regression analysis revealed significant temporal shifts in the age-standardized prevalence of LBP in Pakistan from 1990 to 2021. Between 1990 and 2000, prevalence rose modestly with an APC of 0.09% (95% CI: 0.07 to 0.11, p<0.001). The rate of increase accelerated between 2000 and 2008 (APC: 0.24%, 95% CI: 0.21 to 0.28, p<0.001), followed by an even sharper rise from 2008 to 2011 (APC: 0.60%, 95% CI: 0.32 to 0.88, p<0.001). The most pronounced surge occurred during 2011-2014, with prevalence increasing at 2.30% annually (95% CI: 2.01 to 2.59, p<0.001). However, the trend plateaued between 2014 and 2017 (APC: 0.19%, 95% CI: -0.09 to 0.47, p=0.169), showing no significant change. From 2017 to 2021, prevalence significantly declined at an APC of -0.54% (95% CI: -0.63 to -0.45, p<0.001), marking the first sustained decrease over the study period, as shown in Table 1.

**Table 1:** Joinpoint regression analysis of Prevalence and Years Lived with Disability (YLD) of Low Back Pain in Pakistan, from 1990 to 2021 APC: Annual percent change; CI: confidence interval.

Cohort	Segment	Lower Endpoint	Upper Endpoint	APC	Lower CI	Upper CI	Test Statistic (t)	Prob > |t|
Prevalence	1	1990	2000	0.09*	0.07	0.11	8.58	<0.001
Prevalence	2	2000	2008	0.24*	0.21	0.28	13.8	<0.001
Prevalence	3	2008	2011	0.60*	0.32	0.88	4.54	<0.001
Prevalence	4	2011	2014	2.30*	2.01	2.59	17.22	<0.001
Prevalence	5	2014	2017	0.19	-0.09	0.47	1.45	0.169
Prevalence	6	2017	2021	-0.54*	-0.63	-0.45	-13.01	<0.001
Years Lived with Disability (YLDs)	1	1990	2001	0.08*	0.05	0.11	6.17	<0.001
YLDs	2	2001	2010	0.27*	0.23	0.32	13.19	<0.001
YLDs	3	2010	2015	1.86*	1.73	1.98	30.52	<0.001
YLDs	4	2015	2021	-0.44*	-0.51	-0.38	-13.82	<0.001

Temporal trends in YLDs

From 1990 to 2001, YLDs increased slowly at an APC of 0.08% (95% CI: 0.05 to 0.11, p<0.001). The growth rate intensified between 2001 and 2010, with an APC of 0.27% (95% CI: 0.23 to 0.32, p<0.001). A sharp surge occurred from 2010 to 2015, during which YLDs rose annually by 1.86% (95% CI: 1.73 to 1.98, p<0.001), marking the steepest increase across the study period. However, this upward trajectory was followed by a significant decline between 2015 and 2021, with YLDs decreasing at an APC of -0.44% (95% CI: -0.51 to -0.38, p<0.001), as shown in Table 1.

## Discussion

This study provides the first comprehensive assessment of long-term trends in the burden of LBP in Pakistan from 1990 to 2021, examining both national patterns and provincial disparities. Our examination of GBD data found that YLDs from LBP increased significantly from 1990 to 2015, followed by a minor drop afterward. Furthermore, we found significant geographic variability across provinces, with initially low-burden regions like Punjab and ICT experiencing the greatest relative increases, while traditionally high-burden regions like Gilgit-Baltistan, Khyber Pakhtunkhwa, and Balochistan showed only modest growth. These findings highlight the dynamic and regionally distinct nature of LBP epidemiology in Pakistan.

Temporal trends in YLDs

LBP represents one of the largest contributors to YLDs in Pakistan. According to the GBD 2021 study, LBP accounted for approximately 720 YLDs per 100,000 population in 2021, up from 605 per 100,000 in 1990, a 10.2% rise. This places LBP among the top non-communicable causes of disability nationally, comparable to osteoarthritis and neck pain [1]. Between 1990 and 2001, YLDs due to LBP increased gradually at an APC of +0.08%, followed by an acceleration from 2001 to 2010 (+0.27%). The fastest increase occurred between 2010 and 2015, when the APC increase was +1.86%. This increase is most likely due to demographic changes, such as population expansion, urbanization, and an aging population, as well as lifestyle risk factors including obesity, sedentary behavior, and occupational stress [6,19]. After 2015, YLDs decreased somewhat (APC -0.44%). This decline may be due to multiple factors, including methodological updates in GBD estimation and possible epidemiological changes. Potential contributors could include limited workplace ergonomics initiatives (e.g., occupational health guidelines for manual laborers and healthcare workers introduced during this period), increased availability of physiotherapy services in urban areas, or changes in health-seeking behaviors; however, in the absence of national intervention data, these explanations remain speculative [5]. Similar non-linear temporal trends have been observed worldwide, where advancements in health systems, ergonomic initiatives, and early treatment availability have helped mitigate disability rates despite their persistently high prevalence [20].

Provincial disparities in LBP prevalence

In 1990, northern provinces like Gilgit-Baltistan and Khyber Pakhtunkhwa had the greatest prevalence, which was most likely due to physically hard labour, hills, and limited access to healthcare [21]. In comparison, Punjab and ICT had the lowest frequency. By 2021, all provinces had increased, although Punjab (+14.8%) and ICT (+11.8%) had the highest relative growth rates. These findings may reflect increased urbanization, sedentary lifestyles, and rising obesity rates in metropolitan areas [22]. In contrast, despite maintaining the highest absolute frequency, Gilgit-Baltistan reported just a 2.4% increase, probably reflecting a plateauing effect after decades of high burden or underreporting due to poor health surveillance [23].

Our findings are comparable with previous research from South Asia, which has indicated consistent but varied LBP prevalence across regions [10,24]. For example, Indian studies have found that urbanization and occupational transitions are related to increased disability from LBP, which is consistent with our findings in Punjab and ICT [25]. Regional disparities in Pakistan emphasise the importance of socioeconomic position, healthcare access, and occupational risk factors.

Clinical and public health implications

The rising prevalence and persistent disability burden of LBP in Pakistan have important clinical and public health implications. LBP is the main cause of disability worldwide, contributing significantly to productivity loss, absenteeism, and healthcare costs [26]. In Pakistan, where health resources are already limited, the increased burden creates new issues. Workplace ergonomics, physical activity promotion, weight management, and early referral to physiotherapy are all significant prevention techniques [27]. Furthermore, primary care providers should be provided with standardized LBP management guidelines to prevent needless imaging, overtreatment, and opioid use [28].

At the provincial level, tailored measures may be required. Punjab and ICT require initiatives to combat urban lifestyle risk factors, whilst Khyber Pakhtunkhwa and Balochistan could benefit from occupational health programs focused on agriculture and manual labor. Improving health system surveillance in underserved areas like Gilgit-Baltistan could help capture the true burden and guide equitable resource allocation.

Risk factor contributions and regional context

According to GBD 2021 estimates, occupational ergonomic factors, high body mass index, and smoking together account for nearly 39% of LBP-related YLDs globally, with occupational exposures alone contributing about 27% of the burden. These proportions suggest that Pakistan’s rising prevalence may be driven by similar risk patterns. Beyond gender, other determinants such as profession, age, and socioeconomic status have been shown in South Asian studies to significantly affect LBP prevalence, with higher rates among manual laborers, older adults, and individuals in lower socioeconomic groups. For example, a meta-analysis from India reported lifetime prevalence rates of 48%-66% in adults, especially high among health-care workers and manual laborers, while a national survey in Bangladesh found adult LBP prevalence around 18%-20% [10,29,30]. These regional data underscore the importance of modifiable risk factors and sociodemographic context when interpreting Pakistan’s LBP trends.

Strengths and limitations

A key strength of this study is the use of comprehensive GBD data with standardized methods, which ensures comparability across time and regions. The application of Joinpoint regression adds robustness by detecting important shifts in trends. Provincial-level analysis further highlights regional disparities in Pakistan, providing valuable insights for localized health policy.

However, some limitations should be noted. This study has several limitations. First, it uses secondary GBD data, which are modeled from multiple sources and may carry uncertainty, especially for provinces with sparse local data. Second, the GBD definition of LBP is broad and does not distinguish between acute, chronic, or specific etiologies; therefore, our estimates may overgeneralize distinct clinical entities. Third, because of the ecological design of GBD-based analyses, associations between risk factors (such as high BMI, occupational exposures, and smoking) and LBP cannot be interpreted as causal. Fourth, our interpretations of provincial disparities in terms of occupation and lifestyle are plausible but speculative, as disaggregated GBD data by profession or socioeconomic status are not available. Where possible, we have referenced local health surveys and labor force studies to provide contextual support, but residual uncertainty remains. These limitations should be considered when interpreting our findings.

## Conclusions

This study examined the long-term burden and trends of LBP in Pakistan from 1990 to 2021 using the GBD data. We found that the absolute number of prevalent LBP cases rose from 5.23 million to 13.93 million over the study period - a 166% increase - and that the age-standardized prevalence and YLD rates also increased, with marked gender and provincial disparities. Joinpoint regression revealed a sharp rise in both prevalence and YLDs up to 2015, followed by a modest but significant decline thereafter. These findings highlight three key points. First, Pakistan’s LBP burden is not only high but rising faster than in some neighboring countries, particularly in provinces with initially lower baseline prevalence, such as Punjab and the Islamabad Capital Territory. Second, much of this burden is potentially attributable to modifiable risk factors such as occupational exposures and high BMI, which aligns with regional evidence from India and Bangladesh. Third, differences by sex, geography, and occupational patterns underscore the need for tailored prevention and rehabilitation strategies.

Future research should link GBD estimates with national health, labor force, and socioeconomic datasets to identify high-risk groups, quantify occupation-specific burdens, and monitor the impact of preventive interventions. Such studies would help determine whether the post-2015 decline reflects genuine epidemiological change or methodological refinements. Without timely action, the growing disability burden of LBP may further strain Pakistan’s health system and workforce productivity.

## References

[1] (2023). Global, regional, and national burden of low back pain, 1990-2020, its attributable risk factors, and projections to 2050: a systematic analysis of the Global Burden of Disease Study 2021. Lancet Rheumatol.

[2] Knezevic NN, Candido KD, Vlaeyen JW, Van Zundert J, Cohen SP (2025). Low back pain. Lancet.

[3] (2023). The Lancet: New study shows low back pain is the leading cause of disability globally. Seattle: Institute for Health Metrics and Evaluation.

[4] Chen S, Chen M, Wu X (2022). Global, regional and national burden of low back pain 1990-2019: a systematic analysis of the Global Burden of Disease study 2019. J Orthop Translat.

[5] Wu A, March L, Zheng X (2020). Global low back pain prevalence and years lived with disability from 1990 to 2017: estimates from the Global Burden of Disease Study 2017. Ann Transl Med.

[6] Hartvigsen J, Hancock MJ, Kongsted A (2018). What low back pain is and why we need to pay attention. Lancet.

[7] Shehzad WA, Pervaiz F, Riaz M, Hafeez A, Batool N, Siddiqui SS (2025). Frequency, severity, and associated risk factors of low back pain among nurses working in public tertiary care hospitals of Rawalpindi. A cross-sectional study. J Back Musculoskelet Rehabil.

[8] Ijaz M, Akram M, Ahmad SR, Mirza K, Ali Nadeem F, Thygerson SM (2020). Risk factors associated with the prevalence of upper and lower back pain in male underground coal miners in Punjab, Pakistan. Int J Environ Res Public Health.

[9] Siddiqui AS, Javed S, Abbasi S, Baig T, Afshan G (2022). Association between low back pain and body mass index in Pakistani population: analysis of the software bank data. Cureus.

[10] Shetty GM, Jain S, Thakur H, Khanna K (2022). Prevalence of low back pain in India: a systematic review and meta-analysis. Work.

[11] Momen Majumder MS, Hakim F, Bandhan IH (2022). Low back pain in the Bangladeshi adult population: a cross-sectional national survey. BMJ Open.

[12] Sany SA, Tanjim T, Hossain MI (2021). Low back pain and associated risk factors among medical students in Bangladesh: a cross-sectional study. F1000Res.

[13] Sharma S, Traeger AC, Maher CG, Bista B, Dhimal M, Dixit LP, Sharma S (2025). Prevalence of low back pain in Nepal: results from a nationally representative WHO STEPS survey. J Pain.

[14] (2024). Global incidence, prevalence, years lived with disability (YLDs), disability-adjusted life-years (DALYs), and healthy life expectancy (HALE) for 371 diseases and injuries in 204 countries and territories and 811 subnational locations, 1990-2021: a systematic analysis for the Global Burden of Disease Study 2021. Lancet.

[15] (2024). Global burden and strength of evidence for 88 risk factors in 204 countries and 811 subnational locations, 1990-2021: a systematic analysis for the Global Burden of Disease Study 2021. Lancet.

[16] Hoy D, March L, Brooks P (2014). The global burden of low back pain: estimates from the Global Burden of Disease 2010 study. Ann Rheum Dis.

[17] Bray F, Guilloux A, Sankila R, Parkin DM (2002). Practical implications of imposing a new world standard population. Cancer Causes Control.

[18] Kim HJ, Fay MP, Feuer EJ, Midthune DN (2000). Permutation tests for joinpoint regression with applications to cancer rates. Stat Med.

[19] Hoy D, Bain C, Williams G (2012). A systematic review of the global prevalence of low back pain. Arthritis Rheum.

[20] Buchbinder R, van Tulder M, Öberg B (2018). Low back pain: a call for action. Lancet.

[21] Rana MA, Ahmed SS, Awan N, Siddique N (2024). Are we straining to succeed? Prevalence of work-related musculoskeletal disorders among dentists in teaching hospitals. J Pak Med Assoc.

[22] Ng M, Fleming T, Robinson M (2014). Global, regional, and national prevalence of overweight and obesity in children and adults during 1980-2013: a systematic analysis for the Global Burden of Disease Study 2013. Lancet.

[23] Asif AF (2017). Healthcare challenges in Gilgit Baltistan: the way forward. Pak J Public Health.

[24] Hoy DG, Smith E, Cross M (2014). The global burden of musculoskeletal conditions for 2010: an overview of methods. Ann Rheum Dis.

[25] Shokri P, Zahmatyar M, Falah Tafti M (2023). Non-spinal low back pain: global epidemiology, trends, and risk factors. Health Sci Rep.

[26] Dagenais S, Caro J, Haldeman S (2008). A systematic review of low back pain cost of illness studies in the United States and internationally. Spine J.

[27] Foster NE, Anema JR, Cherkin D (2018). Prevention and treatment of low back pain: evidence, challenges, and promising directions. Lancet.

[28] Qaseem A, Wilt TJ, McLean RM (2017). Noninvasive treatments for acute, subacute, and chronic low back pain: a clinical practice guideline from the American College of Physicians. Ann Intern Med.

[29] Shen L, Cao W, Yu Y (2025). The burden of low back pain in BRICS: an analysis for the global burden of disease study 2021. Front Public Health.

[30] Rahman MA, Alam F, Chowdhury MR ( 2025). Risk factors associated with low back pain in Bangladesh: A cross-sectional study conducted in 2023. Health Sci Rep.

